# Bioluminescent imaging of ABCG2 efflux activity at the blood-placenta barrier

**DOI:** 10.1038/srep20418

**Published:** 2016-02-08

**Authors:** Jeyan S. Kumar, Bih-Rong Wei, James P. Madigan, R. Mark Simpson, Matthew D. Hall, Michael M. Gottesman

**Affiliations:** 1 Laboratory of Cell Biology, Center for Cancer Research, National Cancer Institute, National Institutes of Health, Bethesda, MD 20892, U.S.A.; 2 Laboratory of Cancer Biology and Genetics, Center for Cancer Research, National Cancer Institute, National Institutes of Health, Bethesda, MD 20892, U.S.A.

## Abstract

Physiologic barriers such as the blood placenta barrier (BPB) and the blood brain barrier protect the underlying parenchyma from pathogens and toxins. ATP-binding cassette (ABC) transporters are transmembrane proteins found at these barriers, and function to efflux xenobiotics and maintain chemical homeostasis. Despite the plethora of *ex vivo* and *in vitro* data showing the function and expression of ABC transporters, no imaging modality exists to study ABC transporter activity *in vivo* at the BPB. In the present study, we show that *in vitro* models of the placenta possess ABCG2 activity and can specifically transport D-luciferin, the endogenous substrate of firefly luciferase. To test ABCG2 transport activity at the BPB, we devised a breeding strategy to generate a bioluminescent pregnant mouse model to demonstrate transporter function *in vivo.* We found that coadministering the ABCG2 inhibitors Ko143 and gefitinib with D-luciferin increased bioluminescent signal from fetuses and placentae, whereas the control P-gp inhibitor DCPQ had no effect. We believe that our bioluminescent pregnant mouse model will facilitate greater understanding of the BPB and ABCG2 activity in health and disease.

The placenta forms the interface between the mother and fetus, and is able to maintain two separate circulatory systems. One of the major functions of the placenta is to form a selective barrier to protect and nurture the developing fetus. The syncytiotrophoblast cells, organized in a layer of cells that comprise the apical surface of the blood-placenta barrier (BPB), line the villous processes of the placenta and form both a physical and electrochemical barrier. The syncytium effectively prevents any paracellular transport of solutes from the mother to the fetus^1^,^2^. Transcellular transport occurs through a variety of transporters that mediate bidirectional transport of specific nutrients and metabolites between mother and fetus^1^.

The efflux transporters of the ATP Binding Cassette (ABC) family play a protective role at the BPB. ABCG2 and P-gp (P-glycoprotein, ABCB1) are expressed on the apical side of the BPB and actively efflux xenobiotics against their concentration gradient back into the maternal blood^2^,^3^,^4^,^5^,^6^. This is achieved by intercepting small molecules as they diffuse into the apical cell membrane and effluxing them back into the lumen (the so-called ‘hydrophobic vacuum cleaner’^7^,^8^). For example, the antidiabetic drug glyburide has been shown to be a substrate of ABCG2, which accounts for its low fetal accumulation and relative safety when administered during a pregnancy^9^,^10^,^11^.

Despite advances in characterization of transporter function at the BPB, there is a paucity of literature on imaging this transporter function *in vivo*. Magnetic resonance imaging (MRI) and ultrasound (U/S) are two of the most commonly used imaging modalities for studying placental structure and function. However, little work on temporal imaging of BPB function and drug transporter activity has been reported. Unadkat and co-workers reported PET imaging of the P-gp substrate [^11^C]verapamil to visualize tissue penetrance in pregnant non-human primates^12^,^13^,^14^. However, it is unlikely that PET imaging of placentae and fetuses would be feasible in a pregnant mouse model given the limitations of PET resolution and signal from surrounding organs^15^.

Bioluminescence imaging (BLI) utilizes D-luciferin and firefly luciferase (fLuc) to generate luminescence that is detected by a charge-coupled device (CCD)^16^. Recently, D-luciferin was demonstrated to be a specific substrate of ABCG2, and was used to image the transporter function of ABCG2 at the blood-brain barrier (BBB)^17^. By inhibiting ABCG2 with a small molecule inhibitor, D-luciferin accumulation in the brain increased, resulting in enhanced bioluminescence from mice expressing fLuc in the brain^17^. We hypothesized that it would be possible to design an analogous animal imaging modality to image the role of ABCG2 at the BPB.

To demonstrate ABC transporter expression and function at the BPB, a number of fluorescent substrates and inhibitors for ABCG2, P-gp, and MRP1 were allowed to accumulate in choriocarcinoma cells and primary human villous trophoblasts. Transporter expression was assessed using Western blotting and flow cytometry. A baculovirus (Bacmam) expressing firefly luciferase (fLuc) was also utilized to examine ABCG2 function *in vitro* in choriocarcinoma cells *via* bioluminescence. Finally, a mouse model was designed by mating a transgenic male expressing fLuc with a wild-type female to generate pregnant mice carrying hemizygous pups and placentae expressing fLuc. ABCG2 function was measured in this mouse model, utilizing bioluminescence imaging with luciferin and inhibitors of the transporter. The utility of this imaging model for assessing BPB integrity is discussed.

## Results

### *In vitro* models of the BPB express ABCG2

**Figure 1:**
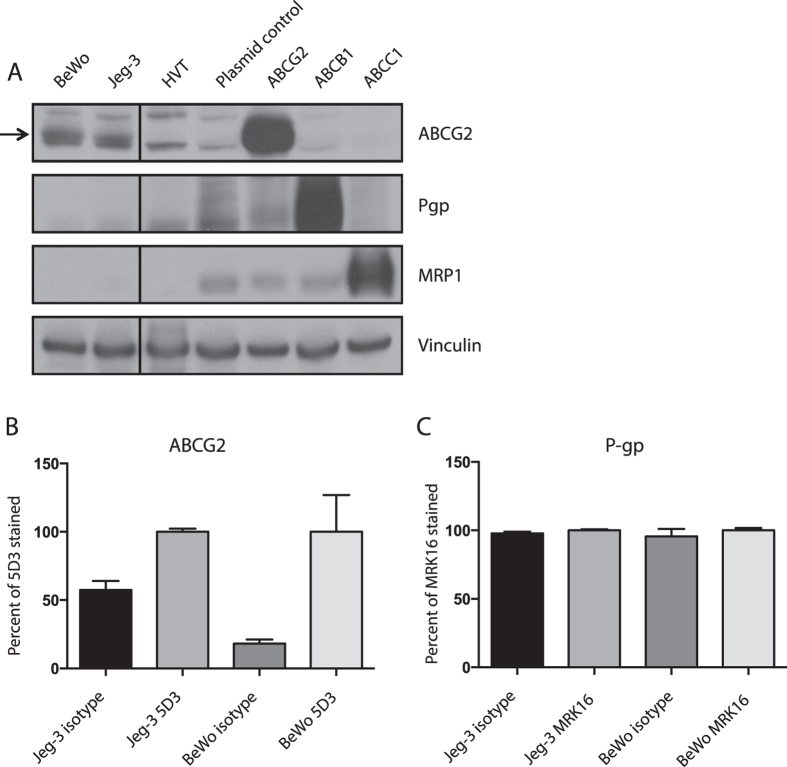
Choriocarcinoma and HVT cell lines express ABCG2 but not P-gp or MRP1. (**A**) Western blot for ABC transporters P-gp, ABCG2, and MRP1 in BeWo, Jeg-3, and HVT cell lysates and positive control HEK cells expressing ABCG2, P-gp, or MRP1. (**B**) Flow cytometry antibody staining of ABCG2. (**C**) Flow cytometry antibody staining of Pgp. Data normalized to fluorescence measured in the antibody stained condition for each cell line from three experiments ± SD (*p < 0.05, **p < 0.01 by Student’s t test).

We first examined the human choriocarcinoma cell lines Jeg-3 and BeWo, and a human primary villous trophoblast (HVT) cell line for ABC transporter activity and expression. Lysates from all three cell lines demonstrated ABCG2 protein expression by Western blot, but not P-gp or MRP1 expression (Fig. 1A). Flow cytometry with intact cells revealed that choriocarcinoma cells, but not HVT, demonstrated cell-surface ABCG2 expression (p < 0.01; Fig. 1B and Fig. S1A). All three of the tested cell lines lacked P-gp (Fig. 1C and Fig. S1B).

To measure ABCG2 transporter function we utilized cytoxicity assays in the presence or absence of specific inhibitors and fluorescent accumulation of substrates. Cytotoxicity assays (Table 1) with the ABCG2 substrate SN-38 (ABCG2) revealed that Jeg-3 and BeWo cells were sensitized to the ABCG2 substrate SN-38 in the presence of the ABCG2 inhibitors Ko143 and FTC (uninhibited IC_50_ 351 ± 72 and 15 ± 3; inhibited IC_50_ 54 ± 4 and 3 ± 0.4, respectively, for Ko143, p < 0.0001). Neither of the cell lines protected against the P-gp substrate paclitaxel, and only BeWo cells showed low-grade MRP1 activity (p < 0.05).

**Figure 2:**
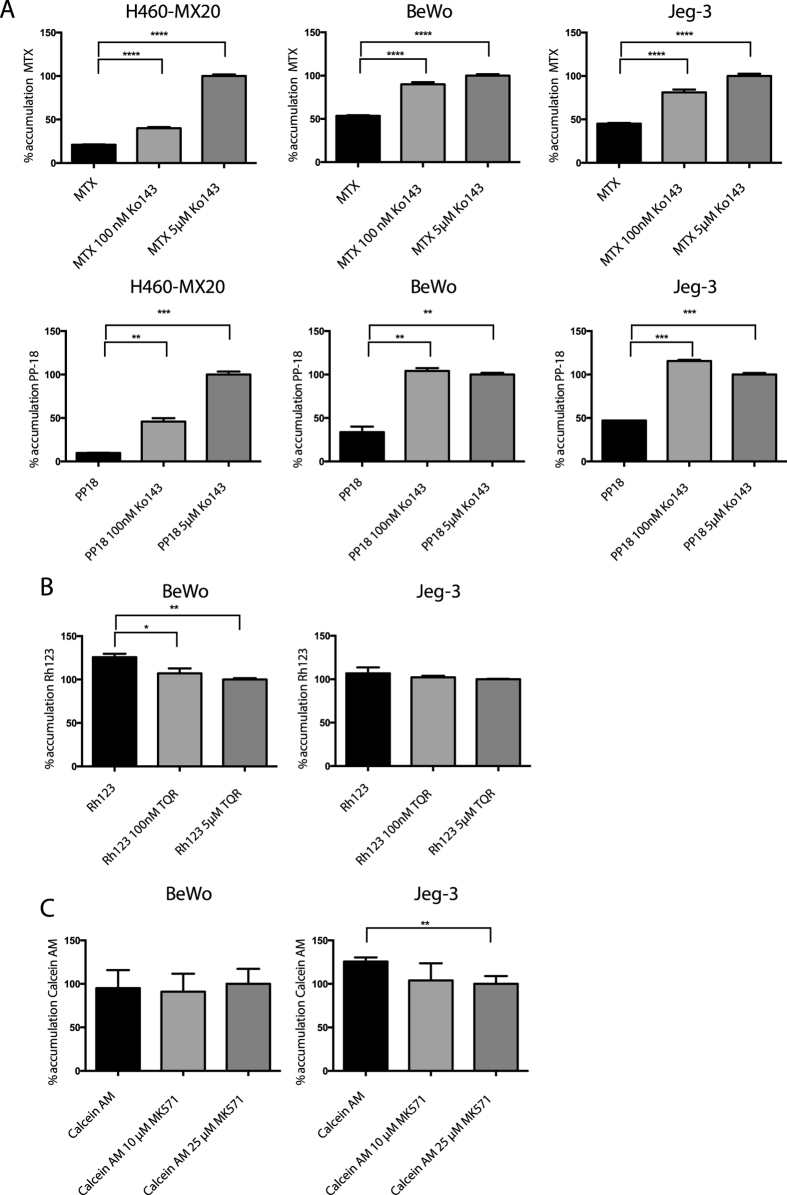
Accumulation of fluorescent substrates of ABCG2, P-gp, and MRP1, measured by flow cytometry. (**A**) Choriocarcinoma cells and positive control ABCG2-expressing cell line H460-MX20 were incubated with ABCG2 substrates mitoxantrone (10 μM) or purpurin 18 (15 μM) with and without Ko143 (100 nM, 5 μM). (**B**) Choriocarcinoma cell lines incubated with P-gp substrate Rhodamine 123 (2 μM) with and without P-gp inhibitor tariquidar (100 nM, 5 μM). (**C**) Choriocarcinoma cell lines incubated with MRP1 substrate Calcein-AM (1 μM) with and without MRP1 inhibitor MK571 (10 μM, 25 μM). All accumulation values are normalized to accumulation of the maximally inhibited condition. Data represent means ± SD of three experiments (**p < 0.01, ***p < 0.001, ****p < 0.0001 by Student’s t test).

ABC transporter activity in BPB cells was directly assessed by measuring cellular accumulation of fluorescent substrates by flow cytometry (Fig. 2 and Fig. S1). All four tested cell lines showed low accumulation, normalized to accumulation in cells exposed to 5 μM Ko143, of the ABCG2 substrates mitoxantrone (21.1 ± 0.3%, 53.5 ± 0.7%, 45.1 ± 0.8%, 83.4 ± 0.4%) and purpurin 18 (9.9 ± 0.2%, 33.8 ± 6.3%, 47.1 ± 0.1%, 87.1 ± 1.8%) for H460-MX20, an ABCG2-overexpressing cell line (positive control), BeWo, Jeg-3, and HVT cells, respectively. The P-gp inhibitor tariquidar did not increase the accumulation of the P-gp substrate rhodamine 123 (Fig. 2B), and the MRP1 inhibitor MK571 did not increase accumulation of the MRP1 substrate calcein-AM (Fig. 2C).

### ABCG2-mediated efflux of D-luciferin regulates bioluminescence in BPB cells

**Figure 3:**
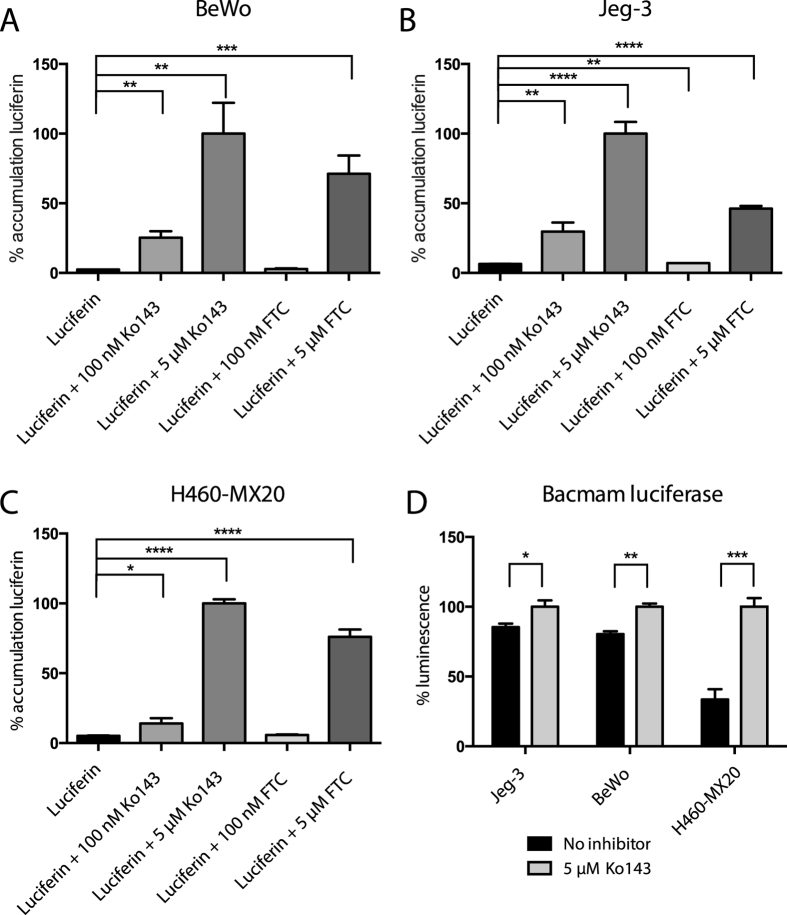
ABCG2 inhibition increases D-luciferin accumulation and bioluminescent signal in choriocarcinoma cells. (**A**) D-luciferin (2 mM) accumulation in BeWo cell line, with and without ABCG2 inhibitors Ko143 (100 nM, 5 μM) and fumitrogen C (FTC; 100 nM, 5 μM) measured by flow cytometry. (**B**) D-luciferin (2 mM) accumulation in Jeg-3. (**C**) D-luciferin (2 mM) accumulation in H460-MX20. (**D**) To measure the effect of ABCG2 on bioluminescence, all three cell lines were transiently transduced with a baculovirus (Bacmam) containing firefly luciferase. Bioluminescence with and without 5 μM Ko143 is reported. All values are normalized to the 5 μM Ko143 condition. Data represent means ± SD of three experiments (*p < 0.05 **p < 0.01, ***p < 0.001, ****p < 0.0001 by Student’s t test).

Given that the specific ABCG2 substrate D-luciferin was to be utilized in the proposed BPB imaging model, we measured its accumulation and the impact of ABCG2 inhibition in the BPB cell lines and H460-MX20 cells. First we took advantage of D-luciferin’s inherent fluorescence^18^ (ex = 350 nm, emm = 530 nm) (Fig. 3A–C and Fig. S3A). In all cases, accumulation of D-luciferin was relatively low (5.2 ± 0.2%, 2.4 ± 0.03%, 6.5 ± 0.1%, 10.2 ± 0.7 for H460-MX20, Bewo, Jeg-3, and HVT cells, respectively), and increased with incubation of Ko143. When cells were co-incubated with another ABCG2 inhibitor, FTC, higher concentrations (5 μM) were required to increase D-luciferin accumulation in all tested cell lines. To assess whether the elevation of D-luciferin accumulation in ABCG2-inhibited cells would result in elevated bioluminescence signal, cells were transiently transduced with fLuc, using an fLuc-containing baculovirus capable of expression in mammalian cells (the so-called ‘BacMam’ system). Consistent with D-luciferin accumulation data, bioluminescence from fLuc-transduced cells exposed to D-luciferin was elevated in the presence of Ko143 (Fig. 3D and Fig. S3B).

### Pregnant mouse model development for bioluminescence imaging

**Figure 4:**
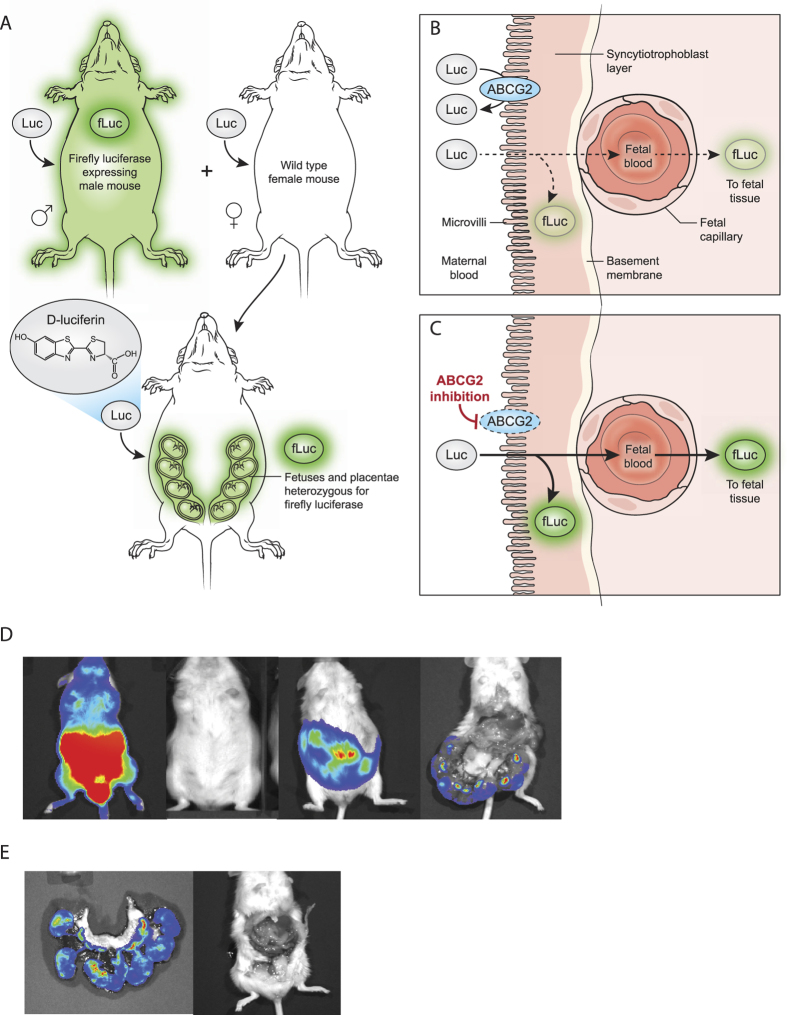
*In vivo* bioluminescent pregnant mouse model. (**A**) Illustration of the breeding strategy to generate pregnant mice carrying placentae and fetuses expressing fLuc. (**B,C**) Diagrammatic representation of the BPB barrier with the hypothesized distribution of d-luciferin (**B**) before and (**C**) after ABCG2 inhibition. (**D**) Bioluminescence images (left to right) of a male CAG-fLuc mouse, non-pregnant female FVB mouse, pregnant FVB mouse, and pregnant FVB mouse imaged immediately post euthanasia with peritoneum opened to visualize uterus, all after being administered 5 mg/kg D-luciferin i.p. for 20 min. (**E**) Bioluminescence images from (left to right) of intact uterus demonstrating BLI signal from fetuses and placentae, and female mouse after uterus was removed with no signal seen, after 5 mg/kg IP d-luciferin for 20 minutes.

We next turned to an *in vivo* bioluminescent mouse model to test ABCG2 function at the BPB. Male FVB mice carrying the CAG-luc-eGFP L2G85 transgene, which results in widespread fLuc expression^19^,^20^, were mated with FVB wild-type female mice. The result was to generate pregnant WT mice carrying placentae and fetuses that are hemizygous for fLuc (Fig. 4A–C). To confirm that bioluminescence was strictly derived from the placenta and fetus, anesthetized mice were injected with D-luciferin using care to avoid inadvertent penetration of abdominal viscera (5 mg/kg, i.p.) and imaged after 20 min (Fig. 4D). Then the uterus was rapidly excised (<1 min) and mothers were reimaged. No signal was observed from the pregnant animals following removal of the uterus (Fig. 4E). Blood drawn antemortem from pregnant mice and incubated with D-luciferin did not produce bioluminescence (not shown), suggesting that the level of fetal cells in the maternal circulation was below the level of bioluminescence imaging (BLI) detection.

**Figure 5:**
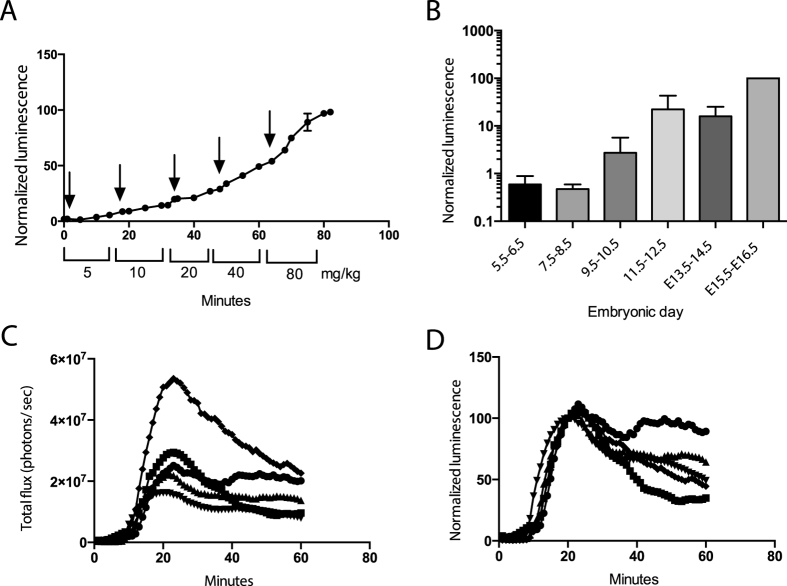
Validation of *in vivo* mouse model. (**A**) Dose escalation study of pregnant mice, carrying fLuc-expressing fetuses and placentae, receiving increasing dosages of D-luciferin (arrow) over 80 minutes. Data normalized to the maximum signal of each mouse and represent mean ± SD of at least three different animals. (**B**) Maximum bioluminescence signals measured every two days between embryonic day 5.5 and 16.5. Data are normalized to the maximum BLI signal measured at the last time point, and are represented as mean ± SD of at least three different animals. (**C**) Bioluminescence signal intensities represented in photons per sec. (**D**) Data presented in (**C**) normalized to the 20-minute time point. Data represent mean ± SEM of five different animals. Number of mice, (**A**) n = 3, (**B**–**D**) n = 4, except for E5.5–6.5 (n = 2).

To determine the optimum dose of D-luciferin for our *in vivo* studies, we injected three mice with serial doses of D-luciferin from 5 to 80 mg/kg (Fig. 5A). A baseline bioluminescent signal was observed following the first injection (5 mg/kg), and signal increased with each dose increase over the course of imaging. As the relatively low 5 mg/kg gave rise to low signal, this dose was chosen for subsequent studies because it provided a relatively low baseline signal. Next, individual pregnant mice were serially imaged every two days between embryonic day 5.5 and 16.5 over 60 min following D-luciferin injection (5 mg/kg, i.p.). Data were normalized to the maximum total flux (photons/sec) of each animal measured during the final imaging session (Fig. 5B). Over the 21-day gestation period, bioluminescence steadily increased until delivery, and no adverse side effects were observed in study mice as a result of anesthesia or D-luciferin administration. (Note that the 5 mg/kg dose used here is lower than the 150 mg/kg dose utilized in BLI protocols.)

### ABCG2 functional role at the BPB

We hypothesized that if BPB ABCG2 was inhibited we would observe an increase in BLI signal. Due to the dynamic changes occurring at the placenta over time, and the variability in the number of fetuses and placentae in each pregnancy, it was necessary to devise an imaging strategy by which bioluminescence could be measured before and after ABCG2 inhibition in each mouse during each imaging experiment. To illustrate the variability between animals, five mice were injected with D-luciferin (5 mg/kg, i.p.) and serially imaged for 60 min (Fig. 5C). Time-activity curves for each mouse were then normalized at the 20 min time point (Fig. 5D), when BLI signal had stabilized.

**Figure 6:**
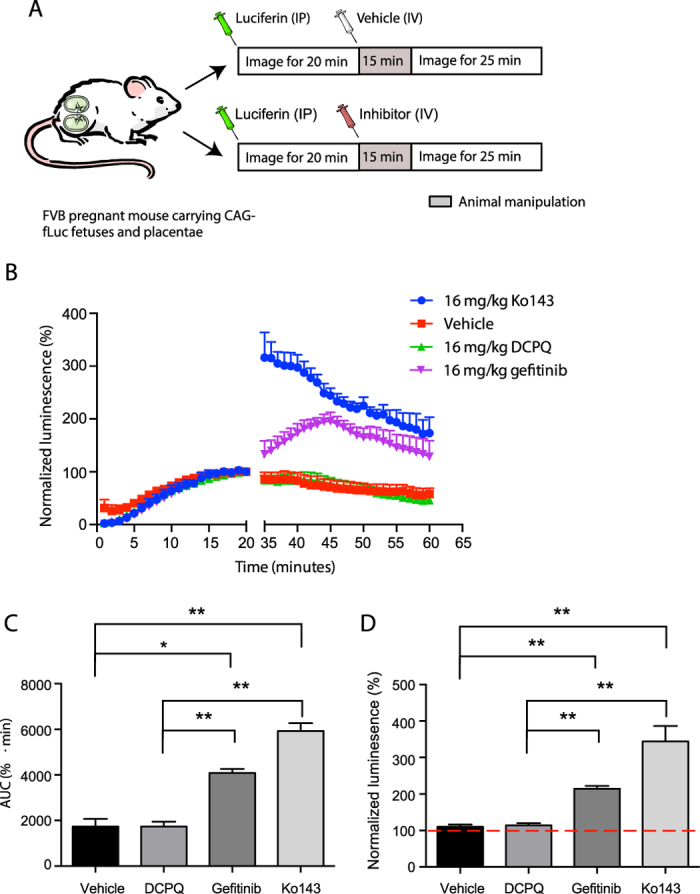
ABCG2 inhibition increases D-luciferin-mediated bioluminescence signal *in vivo.* (**A**) Injection strategy utilized to study the effect of inhibitors. A baseline signal is gathered for the first 20 minutes, when the signal plateaus. At this point mice are removed from the imager and injected with vehicle, or inhibitors, after which they are returned for an additional 25 minutes of imaging. (**B**) Time activity curves of total flux (photons per sec) of pregnant mice, carrying fetuses and placentae expressing fLuc, injected with 5 mg/kg D-luciferin i.p., plus vehicle (negative control), Ko143, Gefitinib, or DCPQ (negative control). All inhibitors were injected at a dosage of 16 mg/kg. Data normalized to the 20-minute time point of each animal (baseline signal). (**C**) AUC acquired from the time activity curves for vehicle control and all three inhibitors. (**D**) Maximum BLI signals acquired from the post-injection phase from all four treatment injection conditions. Data represent mean ± SEM of at least four different animals (*p < 0.05, **p < 0.001 by Student’s t test). Number of mice, (**B–D**) Vehicle n = 5, Ko143 n = 5, DCPQ n = 6, gefitinib n = 4.

The imaging protocol adopted (Fig. 6A) was to inject pregnant mice (Day 12–18) with D-luciferin (5 mg/kg, i.p.), monitor by BLI for 20 min, (at which point signal reached a plateau), after which mice were injected with various inhibitors or vehicle (i.v.), and reimaged during an additional 25 min. When mice were injected with the ABCG2 inhibitors, 16 mg/kg of Ko143 or gefitinib (Fig. 6B), bioluminescence (AUC) increased (6923 ± 1394, 4488 ± 236, p < 0.001, respectively) compared to vehicle control (2932 ± 1111) (Fig. 6C). To confirm that this effect was limited to ABCG2 inhibition, we tested the specific P-gp inhibitor DCPQ (16 mg/kg i.v.), with no change (AUC) in bioluminescence (2421 ± 479; NS) compared to vehicle control. When maximum values were averaged from the post-injection time point (Fig. 6D), gefitinib- and Ko143-treated animals had significantly (p < 0.05) higher bioluminescence compared to DCPQ-treated and vehicle control mice. We and others have shown by positron emission tomography that a DCPQ dose of 16 mg/kg fully inhibits P-gp at the blood-brain barrier^21^, without affecting ABCG2 function^22^.

## Discussion

The aim of this study was to devise an *in vivo* mouse model to study ABCG2 function at the BPB. Our data confirm that placental barrier cells express ABCG2 and possess ABCG2-mediated cellular efflux activity. The cell lines were able to efflux D-luciferin, and ABCG2 inhibition increased D-luciferin accumulation and bioluminescence. We then designed a mouse model to test ABCG2 activity that involved a wild-type female mouse carrying pups and their placentae that expressed fLuc.

We hypothesized that when the pregnant mouse was injected with D-luciferin, ABCG2 at the BPB would efflux most D-luciferin back into the maternal blood, but that a baseline level of D-luciferin would traverse that barrier, interact with embryonic/fetal fLuc-expressing cells, and produce bioluminescence. We designed the model so that all significantly measureable BLI signal arose from the pups and their placentae, by controlling for ‘background noise’ from the host. We further hypothesized that when BPB ABCG2 was inhibited at the BPB, D-luciferin accumulation in the placentae and fetuses would increase due to compromised constitutive efflux transport, giving rise to a greater BLI signal. Consistent with our *in vitro* validation, the mouse model confirmed that ABCG2 inhibition results in increased bioluminescent signal from fetuses and placentae expressing fLuc over baseline, confirming the functional role of ABCG2 at the BPB.

*In vivo* bioluminescence assays have been extensively utilized to measure a variety of biological processes such as tumor burden and gene expression *in vivo*^23^,^24^,^25^. We previously reported on the concept of utilizing bioluminescence to measure ABCG2 function at the blood-brain barrier (BBB)^17^. In that work it was shown that D-luciferin is a specific substrate for both human and mouse ABCG2 among ABC transporters expressed at the BBB. Using a GFAP-fLuc transgenic mouse that expressed fLuc primarily in the brain (GFAP is a marker of astrocytes), low baseline BLI was demonstrated, which increased when D-luciferin was injected in combination with ABCG2 inhibitors including Ko143 and gefitinib, but not when treated with inhibitors of P-gp. The direct observation that ABCG2 plays a role in protecting against single agents at the BBB stimulated an interest in BPB function. It should be noted that while this work was in preparation, Cao *et al.* developed a mouse model to study embryo-fetal exposure to agents administered intravaginally^26^. They crossed a β-actin-luc transgenic male mouse with a WT female to generate pups and placentae expressing fLuc, and observed bioluminescence after administering intravaginal D-lucferin. This model provided insight into the communication of the vagina and developing embryo, but the study was not designed to give insight into the BPB or ABCG2 function at the BPB, and D-luciferin was not administered systemically.

We confirmed in our previous work that ABCG2 inhibitors such as Ko143 and gefitinib did not affect fLuc enzymatic function directly, and so increased signal was not due to off-target interactions. In a separate study, we reported that mouse and human ABCG2 show near-identical recognition of a range of ABCG2 substrates, indicating that mice can act as a meaningful model of human ABCG2 function^27^. We recently examined the selectivity of Ko143, driven by a desire to clarify its utility given the increased use of Ko143 *in vivo*^28^. While we found Ko143 does inhibit P-gp at high (μM) concentrations, we showed that these are far above the achievable free plasma concentration with a 16 mg/kg dose.

The BBB model described above demonstrated entry of D-luciferin into an organ, the brain, that was similar in size in each animal, and therefore fLuc expression levels and BLI signals from animals were similar under each condition imaged. A significant challenge in developing a ABCG2 BPB imaging protocol was that the number of pups (and therefore placentae) being carried by each female was different, and that the tissue amount (and therefore fLuc expression) was changing from day-to-day due to fetal development as females progressed to full term pregnancy. Additionally, ABCG2 activity itself has shown gestational dependent changes in rodent models with peak expression occurring on day 14 and 15 for rat and mouse respectively^5^,^29^,^30^ (and similar data exists for P-gp^31^,^32^). Data on gestational BPB expression of *Abcg2* in mice has been suggested to be inconsistent^33^, but Yeboah *et al.* demonstrated that ABCG2 expression increases over the course of gestation^34^. To accommodate these possibly confounding factors, we measured BLI signal from all mice for 20 mins following injection of BLI substrate. Imaging was then interrupted for 15 mins to allow animal manipulation and dosing, including the injection of inhibitors by tail vein. Imaging was then resumed, and upon completion of the imaging session, each animal’s time course BLI signal was normalized to the signal from that animal at 20 min. As such, the effect of excipient, P-gp or ABCG2 inhibitors was assessed based on the ‘baseline’ signal for that same mouse in that imaging session. All imaging in mice was conducted between days 12 and 18 to minimize variability in BPB expression that may occur over the course of pregnancy.

The BPB assay using luciferin reported here is not limited to analyzing substrates of ABCG2. Since a physically intact syncytium is essential to maintain barrier functions, if this physical barrier is abrogated in any way, the ABCG2 pump will not protect the BPB and luciferin will accumulate in the placentae and fetuses. We envisage, therefore, that this model will prove useful for direct imaging of BPB dysfunction arising from a range of pathologies associated with pregnancy. For example, it has been reported that conditions such as gestational diabetes mellitus (GDM) and pre-eclampsia can alter BPB integrity. Several manipulative mouse models, such as dietary or chemical interventions for GDM, exist to study these pathologies, and the mouse model reported here is amenable to be translated for this purpose.

## Materials and Methods

### Chemicals

D-Luciferin was purchased from Gold Biotechnology (St. Louis, MO). (2R)-anti-5-f3-[4-(10,11-dichloromethanodibenzo-suber-5-yl)piperazin-1-yl]-2-hydroxypropoxygquinoline trihydrochloride (DCPQ) was provided by Victor W. Pike (National Institutes of Mental Health, Bethesda, MD). Gefitinib was purchased from Cayman Chemical (Ann Arbor, MI). Ko143 was purchased from Tocris Bioscience (Bristol, United Kingdom). Tariquidar was purchased from Celon Labs (Hyderabad, India). All other chemicals were purchased from Sigma-Aldrich (St. Louis, MO) unless stated otherwise. Stock solutions of inhibitors were prepared in DMSO, while D-luciferin was prepared in PBS.

### Cell lines

The human choriocarcinoma cell lines Jeg-3 and BeWo were purchased from ATCC and grown in Dulbecco’s Modified Eagle’s Medium (DMEM) and Ham’s F12K Kaighn’s Medium, respectively. HEK 293 cell lines transfected with P-gp, ABCG2, or MRP1 were grown in DMEM supplemented with 1 mg/mL geneticin^35^. Drug-selected H460-MX20 cells that overexpress ABCG2 were grown in RPMI media containing 20 nM mitoxantrone. All media above was supplemented with 10% Fetal Bovine Serum (FBS), penicillin/streptomycin, and 2 mM glutamine. Primary human villous trophoblast (HVT) cells were purchased from Sciencell Research Laboratories and maintained in Trophoblast medium (Sciencell) with included supplements. All cells were incubated at 37 °C in 5% CO_2._ Cells were sub-cultured utilizing a 0.25% trypsin, 9 mM EDTA solution, and were used in experiments between passages 4–20.

### Cytotoxicity assay

Cytotoxicity was measured utilizing the cell viability luminescence assay (CellTiter Glo; Promega; Madison, WI). Cells were plated in 96-well opaque bottom plates (BD-Falcon) at a density of 1,000 cells/well and were allowed to attach overnight before being exposed to the drugs of interest. Stock solutions of cytotoxic drugs SN-38, paclitaxel, and doxorubicin, known to be substrates for ABCG2, P-gp, and MRP1, respectively, with and without their respective inhibitors, were prepared in cell-type specific media. Cells were incubated in serial dilutions of the drugs for 72 h at which point cell growth was assayed as described previously. Luminescence was measured on a Tecan Infinite 200 Pro (Mannedorf, Switzerland) plate reader. Cytotoxicity (IC_50_) was defined as the drug concentration at which the cell viability was reduced to 50% of the untreated control, and was calculated as the mean of three separate observations.

### Flow cytometry

Cellular accumulation of P-gp, ABCG2, and MRP1 fluorescent substrates was measured with a LSR II flow cytometer (BD Biosciences, San Jose, CA). For each experimental condition, 10^6^ cells were suspended in Iscove’s Modified Dulbecco’s Medium (IMDM) supplemented with 5% FBS. Cells were incubated in the dark at 37 °C for 30 minutes with either 2 μM rhodamine 123 (Rh-123), 15 μM purpurin 18 (PP-18), or 500 μM D-luciferin (D-luc), with or without the P-gp inhibitor tariquidar (TQR) or ABCG2 inhibitors Ko-143 and fumitrogen C (FTC) or the MRP1 inhibitor MK571. Cells were then washed and incubated in substrate-free medium with the same inhibitors at 37 °C for 30 min, at which point ice-cold medium was added and the samples centrifuged. Cells were resuspended in ice-cold phosphate buffered saline and kept on ice until flow cytometry (<1 h). Cells were gated for forward versus side scatter and the geometric mean of fluorescence intensity of the substrate was measured for 20,000 events, using excitation/emission wavelengths: D-luciferin (355/530), PP-18 (633/620), Rh-123 (488/530). Fluorescence-activated cellular sorting (FACS) data were analyzed using FlowJo software version 8.7 (Tree Star, Inc.).

Cell surface expression of BCRP was measured by incubating trypsinized cells with anti-ABCG2 5D3-PE monoclonal antibody (eBiosceince, 1 μg per 200,000 cells) at 37 °C for 30 min. Cells were centrifuged, antibody removed, and the fluorescence measured by flow cytometry as described above, with excitation/emission (488/530), and data analyzed using FlowJo software (Tree Star, Inc.). To measure the cell surface expression of P-gp, trypsinized cells were incubated with anti-P-gp MRK-16 primary antibody (Enzo, 1 μg per 200,000 cells) for 40 min. Following this primary antibody was removed and secondary anti-mouse-PE antibody (Enzo, 0.5 μg per 200,000 cells) was added and cells incubated for 30 min. The antibody was removed, cells were washed with PBS, and then analyzed by flow cytometry as described above.

### BacMam cloning and amplification of BacMam fLuc

BacMam baculovirus was generated in the Protein Expression Laboratory at the Frederick National Lab for Cancer Research (FNLCR) as described previously^36^. In brief, the Bac-to-Bac Baculovirus Expression System (Life Technologies) was used according to the manufacturer’s protocol to generate recombinant BacMam baculovirus. Briefly, Gateway cloning was used to transfer fLuc cDNA from bacterial cloning plasmids to the pDest-625 expression vectors for BacMam baculovirus. Expression vectors were transformed into *E. coli* DH10Bac cells where site-specific transposition of the gene of interest into a baculovirus shuttle vector (bacmid) occurred. Blue-white screening was carried out, white colonies were selected, and bacmid DNA was purified by alkaline lysis. Bacmid DNA was transfected into insect cells to generate recombinant BacMam baculovirus.

### BacMam fLuc transduction and bioluminescence

The BacMam-fLuc virus was added to 2.5 × 10^6^ cells at a titer of 1:50 viral particles per cell, unless otherwise specified, in 3 mL of media and incubated for 1hr at 37 °C. 20 mL of media were subsequently added, and cells were plated into 96-well opaque bottom plates (BD-Falcon) at a density of 10,000 cells/well and incubated for 24 h in a 37 °C incubator. Media were then aspirated and 100 μL luciferin, with and without Ko143, dissolved in IMDM media with 5% FBS was added. Plates were read on a Tecan Infinite 200 Pro plate reader.

### Western blot analysis

Protein expression levels of the ABC family drug transporters, ABCB1, ABCG2, and ABCC1, were assessed via Western blot analysis. Cellular protein lysates were prepared and resolved by SDS-PAGE, as described previously^37^. In brief, cells were trypsinized and centrifuged after which supernatant was removed and cell pellets were resuspended in 100 μL of pre-cooled lysis buffer (10 mM Tris, 0.1% Triton-X 100, 10 mM MgSO_4_, 2 mM CaCl_2,_ 1% aprotinin, 1 mM AEBSF (Tocris), 2 mM DTT and 20 μg/mL micrococcal endonuclease (DNAseI)). Resuspended cells were flash frozen on dry ice and subsequently thawed on wet ice. Lysis was achieved by three cycles of a 1-minute sonication followed by a 30-second cool-down on ice, and added to an equivalent amount of 5× sodium dodecyl sulfate (SDS) buffer prior to loading. Lysates were resolved on a 3 to 8% NuPAGE Novex Tris-acetate gel (Invitrogen), and transferred to nitrocellulose membranes (Invitrogen). Membranes were blocked in 20% milk for 30 min at room temperature and subsequently probed for 1 hour at room temperature for expression of ABCB1, ABCG2, ABCC1, and vinculin using C219 (Fujirebio Diagnostics), BXP-53 (Abcam), MRP1 (Enzo Life Sciences) and anti-vinculin [clone hVIN-1] (Sigma-Aldrich) monoclonal antibodies, respectively. Membranes were washed with TBS-T and probed with anti-mouse IgG-horseradish peroxidase (HRP)-conjugated secondary antibody (Cell Signaling Technology) at room temperature for 1 hour, followed by additional washes with TBST. Membranes were incubated in Western Blotting Luminol Reagent (Santa Cruz) and the signal developed on HyBlot ES autoradiography film (Denville Scientific, Inc.).

### Animals

To generate pregnant mice carrying placentae and fetuses capable of producing bioluminescence, 8- to 24-week old male FVB transgenic mice (The Jackson Laboratory) carrying the CAG (human cytomegalovirus immediate early promoter enhancer with chicken beta-actin/rabbit beta-globin hybrid promoter)-luc-eGFP L2G85 transgene were crossed with 6- to 12-week old FVB WT female mice. Mice were utilized for experiments between embryonic day 10 and 18 unless otherwise specified. All animals were maintained under a 12 h light/dark cycle, temperature-controlled environment with free access to food and water at all times. Animal experiments were performed in accordance with the Guide for Care and Use of Laboratory Animals^38^ and were approved by the National Cancer Institute Animal Care and Use Committee.

### Formulations for mice

D-luciferin solutions were prepared in sterile normal saline (NS) at a concentration of 1.5 mg/mL, and stored at −20 °C. A dosage of 5 mg/kg D-luciferin was utilized, as it provided a lower baseline signal from the placentae and fetuses. All inhibitors were used at a dosage of 16 mg/kg and prepared in a vehicle containing dimethyl sulfoxide, propylene glycol, and 0.9% NS, in a 2:2:1 ratio, respectively^39^.

### Bioluminescence imaging

Animals were anesthetized with isoflurane (4% induction, 1.5% maintenance) in O_2_ for the duration of the procedure. Bioluminescent signals were detected using an IVIS100 imager (Perkin Elmer). Intraperitoneal (i.p.) D-luciferin was administered, and mice were serially imaged over a 20 min period. Imaging was then interrupted, and ABCG2 inhibitor or vehicle, was injected intravenously (i.v.), or animals went uninjected, and then were returned to the imager for an additional 30 min. Images were captured in a sequence with 30 s exposure every 60 s. BLI data were analyzed using LivingImage software (PerkinElmer) with the same region of interest (ROI) used for the entire sequence. Background subtraction was applied to each data point.

### Statistical analysis

For *in vitro* studies, data are expressed as mean ± S.D. from three fluorescence efflux assays and from three cytotoxicity assays. After the data were tested for homogeneity of variance, statistical significance was evaluated for fluorescence efflux and cytotoxicity assays utilizing the Student’s t test (unpaired, two-tailed, α = 0.05) and by a two-way analysis of variance followed by the Bonferroni post-t-test (α = 0.05). For *in vivo* BLI studies, data are expressed as mean ± S.E.M. from at least four mice. Total flux (photons per second) was plotted against time (minutes) to construct time activity curves (photons per second 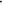 minute). Total area under the curves of time (minutes) vs total flux (photons/sec) was determined by the trapezoidal method using Graphpad Prism 6.0 (La Jolla, CA). After the data were tested for homogeneity of variance, differences in mean AUC (p/s 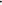 min) were compared using a one-way ANOVA followed by the Bonferroni post-test for multiple comparisons (α = 0.05).

## Additional Information

**How to cite this article**: Kumar, J. S. *et al.* Bioluminescent imaging of ABCG2 efflux activity at the blood-placenta barrier. *Sci. Rep.* **6**, 20418; doi: 10.1038/srep20418 (2016).

## Supplementary Material

Supplementary Information

## Figures and Tables

**Table 1:** Effect of inhibition of ABCG2, P-gp, and MRP1 on cytotoxicity (IC_50,_ nM) of Jeg-3 and BeWo cells.

Cell line	Cytotoxic drug	IC_50_^a^	Cytotoxicity (nM) with inhibitors (1 μM)^a^							
			Ko-143		FTC		TQR		MK571	
			IC_50_	RI^b^	IC_50_	RI^b^	IC_50_	RI^b^	IC_50_	RI^b^
BeWo	SN-38	350.7 ± 71.9	23.8 ± 9.1***	14.7	53.7 ± 3.8**	6.5	–	–	–	–
Jeg-3	SN-38	15.0 ± 2.6	2.8 ± 0.4***	5.4	2.4 ± 0.3***	6.3	–	–	–	–
BeWo	Paclitaxel	2.9 ± 0.8	–	–	–	–	5.0 ± 0.3*	0.6	–	–
Jeg-3	Paclitaxel	7.7 ± 0.3	–	–	–	–	9.7 ± 1.4	0.8	–	–
BeWo	Doxorubicin	141.7 ± 6.1	–	–	–	–	–	–	114.4 ± 9.6*	1.2
Jeg-3	Doxorubicin	21.7 ± 3.7	–	–	–	–	–	–	17.4 ± 3.9	1.2

^a^Data represent three observations ± SD (*p < 0.05, **p < 0.01, **p < 0.001).

^b^Relative Inhibition (RI) is calculated as the ratio of the IC_50_ of the uninhibited cell over the IC_50_ of the inhibited condition.
