# Supplementary material for: Bioluminescent imaging of ABCG2 efflux activity at the blood-placenta barrier
Source: Sci Rep. 2016 Feb 8;6:20418. doi: 10.1038/srep20418 (PMC4745077; doi:10.1038/srep20418)
Supplement: Supplementary Information [file srep20418-s1.pdf]

Supplementary Information

Bioluminescent imaging of ABCG2 efflux activity at the blood-placenta barrier

Jeyan Kumar, Bih-Rong Wei, James P. Madigan, R. Mark Simpson, Matthew D. Hall, Michael M. Gottesman

Supplementary Figure S1

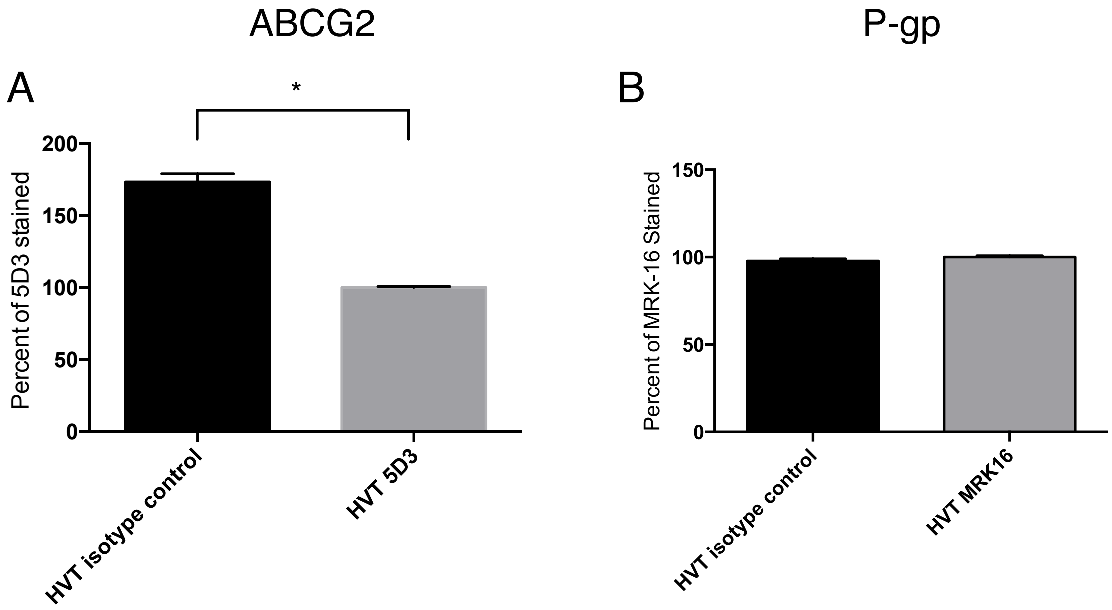

Figure S1. Cell surface expression of transporters by HVT cells. (A) Undetectable levels of ABCG2 expression, as determined by flow cytometry. (B) Undetectable levels of P-gp expression, as determined by flow cytometry. Data normalized to fluorescence measured in the antibody stained condition for each cell line from three experiments ± SD (*$p < 0.05$ by Student's t test).

Supplementary Figure S2

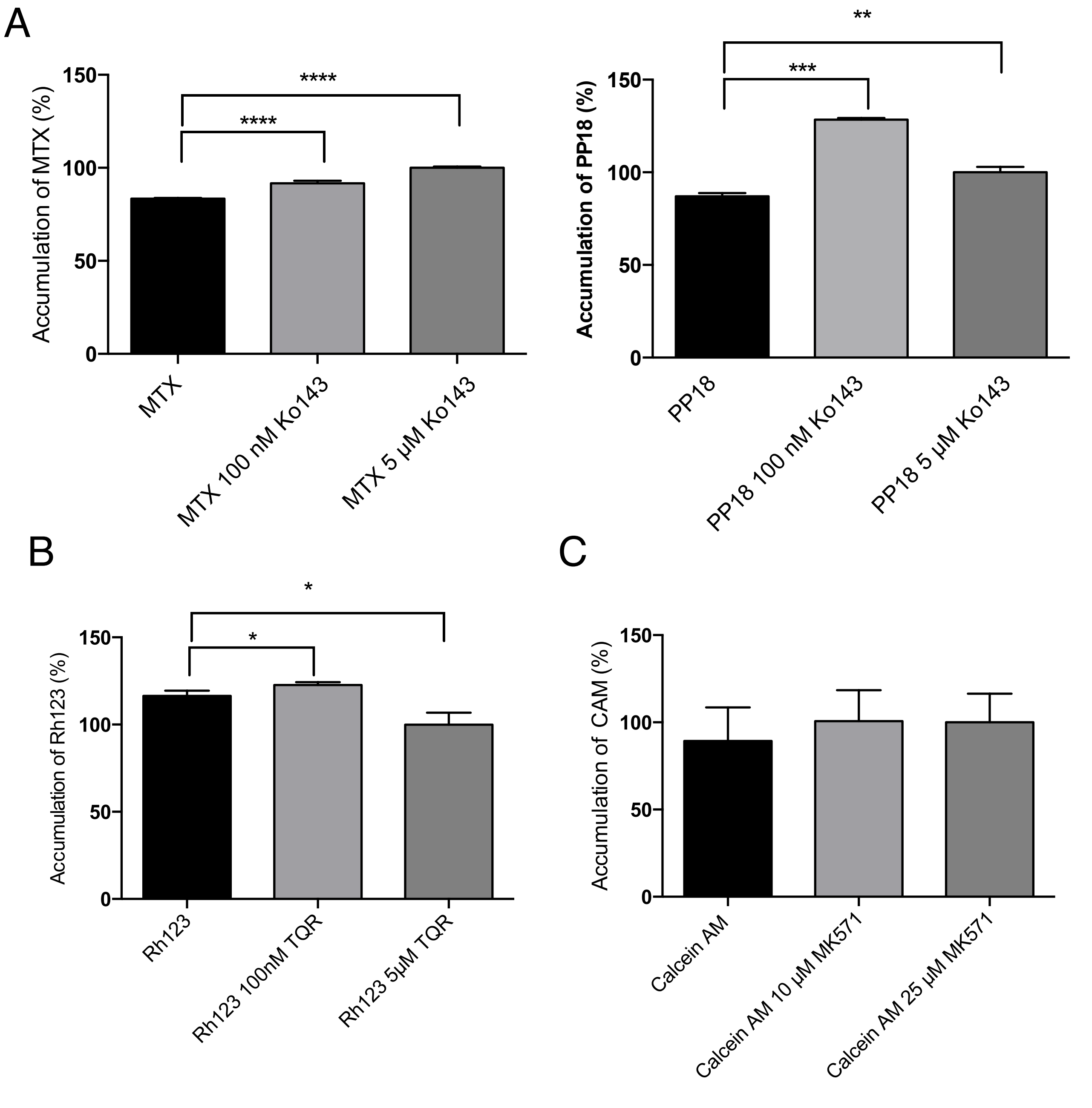

Figure S2. Accumulation of fluorescent substrates of ABCG2, P-gp, and MRP1 measured by flow cytometry in HVT cells. (A) ABCG2 substrates, mitoxantrone (10 µM) and purpurin 18 (15 µM) accumulation increased with Ko143 (100 nM, 5 µM) coincubation. (B) Accumulation of P-gp substrate rhodamine 123 (2 µM) and MRP1 substrate calcein-AM (1 µM) accumulation did not increase with coincubation with their respective inhibitors tariquidar (100nM, 5 µM) and MK571 (10 µM, 25 µM). All accumulation values are normalized to accumulation of the maximally inhibited condition. Data represent means ± SD of three experiments (**$p < 0.01$, ***$p < 0.001$, ****$p < 0.0001$ by Student's t test).

Supplementary Figure S3

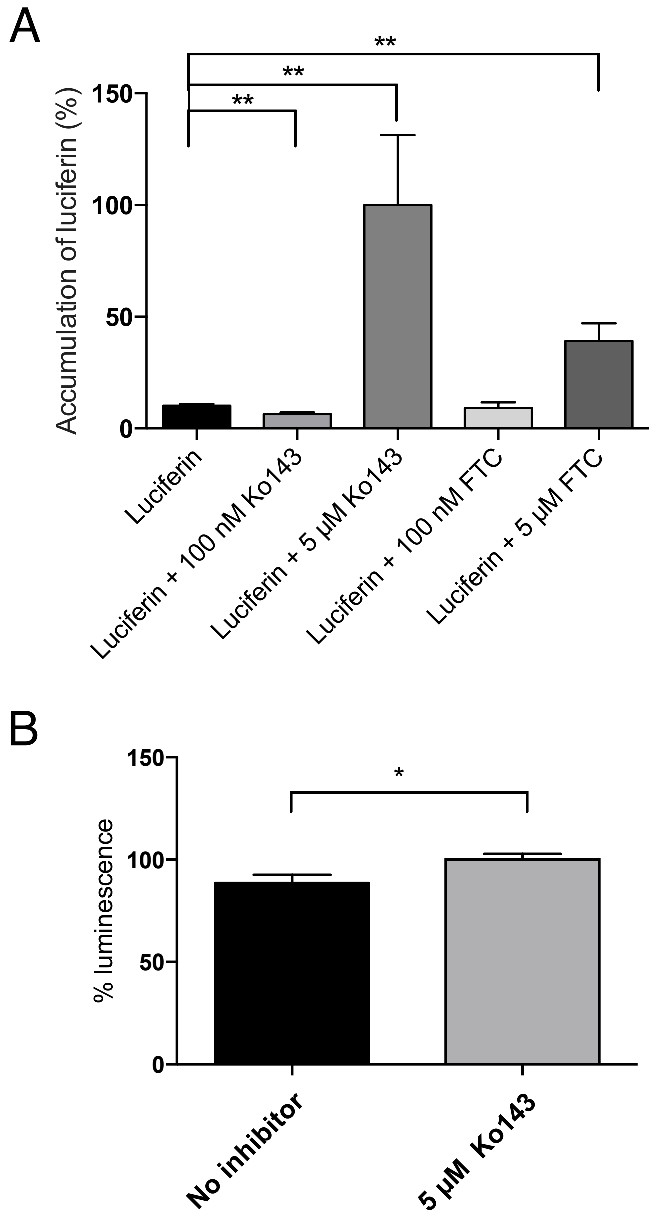

Figure S3. ABCG2 inhibition increases D-luciferin accumulation and bioluminescent signal in HVT cells. (A) D-luciferin (2mM) accumulation in HVT cells increases with ABCG2 inhibitors Ko143 (100nM, 5 µM) and fumitrogen C (FTC; 100 nM, 5 µM) measured by flow cytometry. (B) To measure the effect of ABCG2 on bioluminescence HVT cells were transiently transduced with a baculovirus (BacMam) containing firefly luciferase. Bioluminescence with and without 5 µM Ko143 is reported. All values are normalized to the 5 µM Ko143 condition. Data represent means ± SD of three experiments (*$p< 0.05$, **$p< 0.01$ by Student's t test).
